# Fungal Species Associated with Tuber Rot of Foshou Yam (*Dioscorea esculenta*) in China

**DOI:** 10.3390/jof11050380

**Published:** 2025-05-16

**Authors:** Haifeng Liu, Aye Aye Htun, Sein Lai Lai Aung, Hyunkyu Sang, Jianxin Deng, Yaqun Tao

**Affiliations:** 1 Department of Plant Protection, College of Agriculture, Yangtze University, Jingzhou 434025, China; liuhaifeng14@163.com (H.L.); seinlailaiaung.ppd@gmail.com (S.L.L.A.); 2 State Key Laboratory of High-Efficiency Production of Wheat-Maize Double Cropping, Agronomy College, Henan Agricultural University, Zhengzhou 450046, China; ayeayehtun.yau@gmail.com; 3 Department of Integrative Food, Bioscience and Biotechnology, Chonnam National University, Gwangju 61186, Republic of Korea; hksang@jnu.ac.kr; 4 Wuxue Agricultural Development Center, Wuxue 435400, China

**Keywords:** Foshou yam, tuber rot, fungal pathogens, pathogenicity

## Abstract

Foshou yam (*Dioscorea esculenta*) is a tuber food crop in China. It is a rare species of the yam family and known for its high nutritional value. From 2019 to 2021, tuber rot was observed in Foshou yam in Wuxue, Hubei Province, China. Fungal strains were isolated from diseased tubers, and ten representative strains were identified based on microscopical characterization and multi-locus phylogenetic analysis. A total of five different species were identified, including *Curvularia geniculata*, *Curvularia muehlenbeckiae*, *Fusarium commune*, *Penicillium oxalicum*, and *Penicillium sclerotigenum*. Pathogenicity test revealed that these fungi are the pathogens of tuber rot in Foshou yam. Among them, *P*. *oxalicum* exhibited the strongest pathogenicity. To our knowledge, this is the first report of tuber rot in *D. esculenta* caused by these five species worldwide. This study provides important information for the future management of tuber rot in Foshou yam.

## 1. Introduction

Yam (*Dioscorea* spp.), a perennial tuberous crop, serves as a staple food source in tropical and subtropical regions [1,2]. It ranks as the fourth most produced root and tuber crop globally, following potatoes, cassava, and sweet potatoes, with annual production reaching approximately 89 million tons (2023, FAO, https://www.fao.org/faostat, accessed on 10 May 2025). As one of the primary centers of origin and domestication for yams, China boasts a cultivation history exceeding two thousand years [3,4]. The two predominant cultivated species, *Dioscorea polystachya* and *Dioscorea alata*, are extensively grown throughout China for vegetable crops and traditional medicinal resources [5,6]. Yam tubers contain a diverse range of nutritional ingredients and bioactive compounds, including starch, dietary fiber, proteins, polysaccharides, allantoin, dioscorin, flavonoids, sapogenins, polyphenols, and others [7]. These phytochemicals exhibit pharmacological properties such as spleen and stomach nourishment, immune enhancement, and anti-aging effects [7]. Furthermore, the tuber’s unique flavor and bioactivities have driven substantial consumer demand, positioning yam-derived products as promising candidates for functional food development and nutraceutical applications [7].

Foshou yam (*D. esculenta*) is a rare variety, mainly grown in Wuxue, Hubei Province, China, and known for its unique taste and high nutritional value [8,9]. It has a low soluble sugar content but is rich in amino acids, making it an ideal functional food. In addition, it is colloquially termed the ‘Dabie Mountain Ginseng’, reflecting its esteemed status in traditional health practices [9].

Fungal diseases pose a significant threat to yam production. Many fungal pathogens have been documented to infect yams. For example, yam anthracnose, caused by *Collectotrichum gleosporioides*, is one of the most important diseases in global yam production [10,11]. Concentric leaf spot, caused by *Sclerotium rolfsii*, is considered the second most destructive foliar disease on yam [12]. This pathogen is able to produce extensive hyphae and sclerotia, enhancing its persistence in the field [13]. Many other pathogens, such as *Alternaria alternata*, *Cercospora dioscoreae*, *Curvularia eragrostidis*, *Cylindrosporium dioscoreae*, *Pseudocercospora contraria*, and *Nigrospora oryzae*, have also been reported to cause leaf spot on yam [10,14,15]. Yam tuber rot, caused by various fungal pathogens, is a major postharvest constraint that substantially shortens the storage potential of yam tubers. These pathogens include species belonging to *Fusarium*, *Aspergillus*, *Macrophomina*, *Rhizopus*, *Penicillium*, *Sclerotium*, and *Botrydiplodia*. Among them, *Fusarium* tuber rot is especially damaging [10,16]. Other factors, such as the soil nutrient status, as well as the interactions among plants, endophytes, saprophytes, and pathogens, may also have some impact on plant fungal diseases [17].

*Dioscorea esculenta* is an important yam species in China; however, research on fungal diseases affecting this crop remains limited. Identifying the fungal pathogens associated with *D. esculenta* is essential for effective disease management and sustainable production. Between 2019 and 2021, tuber rot was observed in Foshou yam (*D. esculenta*) in Wuxue, Hubei Province, China. The disease led to significant yield losses, resulting in considerable economic impact. This study aims to isolate and identify the causal pathogens of tuber rot in Foshou yam through morphological and molecular characterization, thereby providing a scientific basis for future disease management strategies.

## 2. Materials and Methods

### 2.1. Fungal Isolation

**Figure 1:**
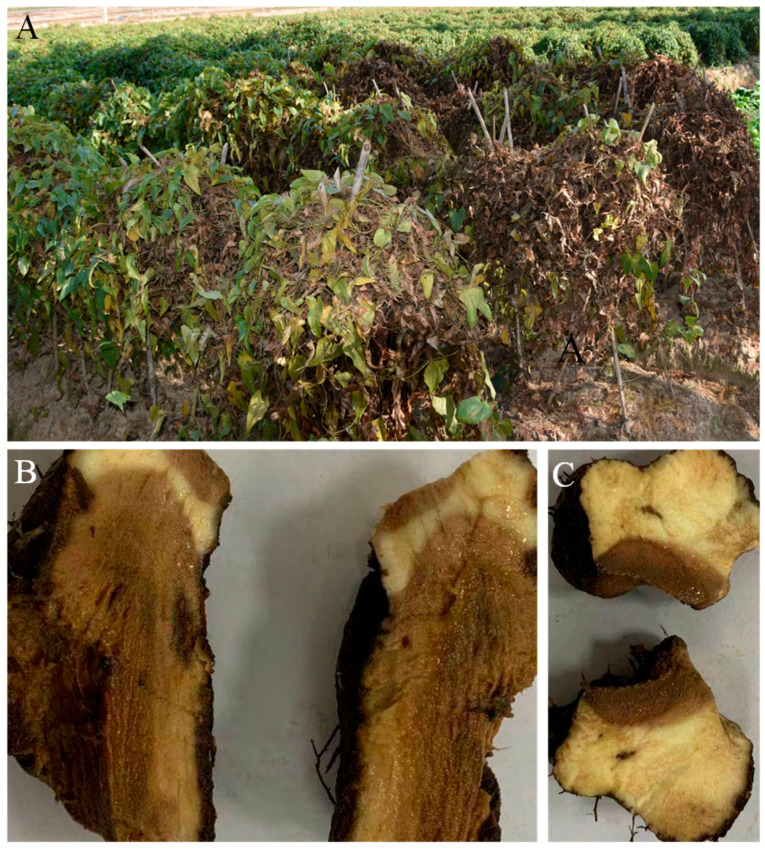
Photographs of diseased Foshou yam (*Dioscorea esculenta*) in Wuxue city, China. (**A**) Diseased Foshou yam plants in the farm; (**B**,**C**) typical symptoms of yam tuber rot (cross-section).

Diseased Foshou yam plants were observed in Wuxue, Hubei Province, China, between 2019 and 2021, exhibiting wilting symptoms (Figure 1A). The tubers showed rot and watery necrotic lesions (Figure 1B,C). Infected tubers were collected in sterile plastic bags and transported to the laboratory. For fungal isolation, tubers were rinsed with running water to remove soil and cut into 3–4 mm pieces using a sterilized blade. The tissues were surface-disinfected in 5% sodium hypochlorite for 5 min, rinsed three times with sterile distilled water, and air-dried in a laminar flow cabinet. The samples were then plated onto potato dextrose agar (PDA; Difco™, Detroit, MI, USA) and incubated at 25 °C in the dark for 3–7 days. Hyphal tips from emerging colonies were sub-cultured three times on fresh PDA to obtain pure isolates, which were preserved in the Fungi Herbarium of Yangtze University, Jingzhou, China.

### 2.2. DNA Extraction and PCR Amplification

To identify the fungi, isolates with distinct colony characteristics were selected as representative strains for genomic DNA extraction and PCR amplification. DNA was extracted from fresh 3-day-old mycelia grown on PDA using a modified CTAB method [18]. Different gene regions, including the internal transcribed spacer (ITS), β-tubulin (*TUB2*), calmodulin (*CAL*), elongation factor 1-α (*TEF1*), glyceraldehyde-3-phosphate dehydrogenase (*GAPDH*), and second largest subunit of the RNA polymerase (*RPB2*) were amplified using primer pairs ITS4/ITS5 [19], Bt2a/Bt2b [20], CMD5/CMD6 [21], EF1-728F/EF1-986R [22], gpd1/gpd2 [23], and RPB2-5F/RPB2-7cR [24], respectively. Different gene regions were chosen for PCR amplification based on fungal genera: ITS and *GAPDH* for *Curvularia* spp., ITS, *TUB2*, *TEF1*, and *RPB2* for *Fusarium* sp., and ITS, *TUB2*, *CAL*, and *RPB2* for *Penicillium* spp. The PCR reaction mixture was prepared in a total volume of 25 μL, consisting of 2 μL DNA template, 1.25 μL of each forward and reverse primer, and 20.5 μL of 1.1 × Taq PCR StarMix (TSINGKE, Beijing, China). The PCR amplification was performed using a Bio-Rad T100^TM^ Thermal Cycler (Hercules, CA, USA). Successful PCR products were sent to BGI (Beijing, China) for purification and Sanger sequencing (both directions).

### 2.3. Phylogenetic Analysis

Nucleotide sequences obtained from BGI were manually checked with BioEdit v7.0.9 [25]. The sequences were then analyzed with BLASTn (https://blast.ncbi.nlm.nih.gov/Blast.cgi, accessed on 5 April 2025) to find similar sequences in the NCBI database. Reliable reference sequences were obtained from recent publications [26,27,28,29,30,31,32]. Phylogenetic analyses were conducted using the OFPT (One-click Fungal Phylogenetic Tool) software v1.9.0 [33]. Specifically, sequences of each genetic region were aligned by MAFFT v7.520 [34] and trimmed by TrimAl v1.2 [35]. Nucleotide substitution models of each dataset were then tested by ModelFinder v1.6.12 [36]. Finally, maximum likelihood (ML) and Bayesian inference (BI) analyses were performed with IQ-TREE v1.6.12 [37] and MrBayes 3.2.7 [38], respectively, using the concatenated datasets with partition information. Sequences generated in this study were deposited in GenBank (https://www.ncbi.nlm.nih.gov/) with accession numbers shown in Table S1.

### 2.4. Morphology

Colony morphology of the fungal strains was examined on different media after incubation at 25 °C in the dark for 7 days. For the species of *Curvularia*, PDA and PCA (potato carrot agar) were used. PDA was also used for the species of *Fusarium*. For the species of *Penicillium*, three different media were used, including malt extract agar (MEA), Czapek yeast extract agar (CYA), and yeast extract sucrose agar (YES). Conidia morphology of the fungal species was examined under an ECLIPSE Ni-U microscope (Nikon, Tokyo, Japan). Conidial characteristics, such as shape and dimensions, were recorded and compared with the descriptions in previous publications.

### 2.5. Pathogenicity Test

Pathogenicity of the strains was conducted on healthy Foshou yam tubers. Prior to the assay, the tubers were washed and surface-disinfected as previously described. Each tuber was then bored with a sterile 6 mm cork borer. To perform the assay, fungal strains were cultured on PDA for 5–7 days. Mycelia plugs (5 mm diam) taken from the edge of the colonies were placed in the holes after scooping out the yam tissue. The scooped-out tissue was used to cover the surface of the inoculation points. Tubers in control group were inoculated with pure PDA plugs. The wounds were sealed with petroleum jelly, and the inoculated tubers were placed in a sterile plastic bag. The pathogenicity test was repeated three times. Diseased tubers were used for re-isolation to confirm the causal fungi in accordance with Koch’s postulates.

### 2.6. Statistical Analysis

Diameters of tuber lesions in pathogenicity assays were measured. Statistical significance was determined by the least significant difference (LSD) test (*p* ≤ 0. 05) in R software (2019, R Core Team, Vienna, Austria).

## 3. Results

### 3.1. Fungal Strains

In this study, diseased tubers of Foshou yam collected from Wuxue City were used for fungal isolation. Based on colony morphology, 10 representative fungal strains were selected for further analysis. The strain numbers were YZU 191548, YZU 191566, YZU 201358, YZU 211038, YZU 211044, YZU 201352, YZU 201354, YZU 201362, YZU 211048, and YZU 211050.

### 3.2. Molecular Phylogeny

Based on BLASTn searches (e-value ≤ 0.05), strains YZU 191548 and YZU 191566 showed 99–100% similarity to *Curvularia* species. Phylogenetic analysis of the genus *Curvularia* was conducted using 80 strains (including the two from this study), based on concatenated sequences of ITS and *GAPDH*. The best-fit substitution models for ITS and *GAPDH* were TNe+R2 and TNe+I+G4, respectively.

**Figure 2:**
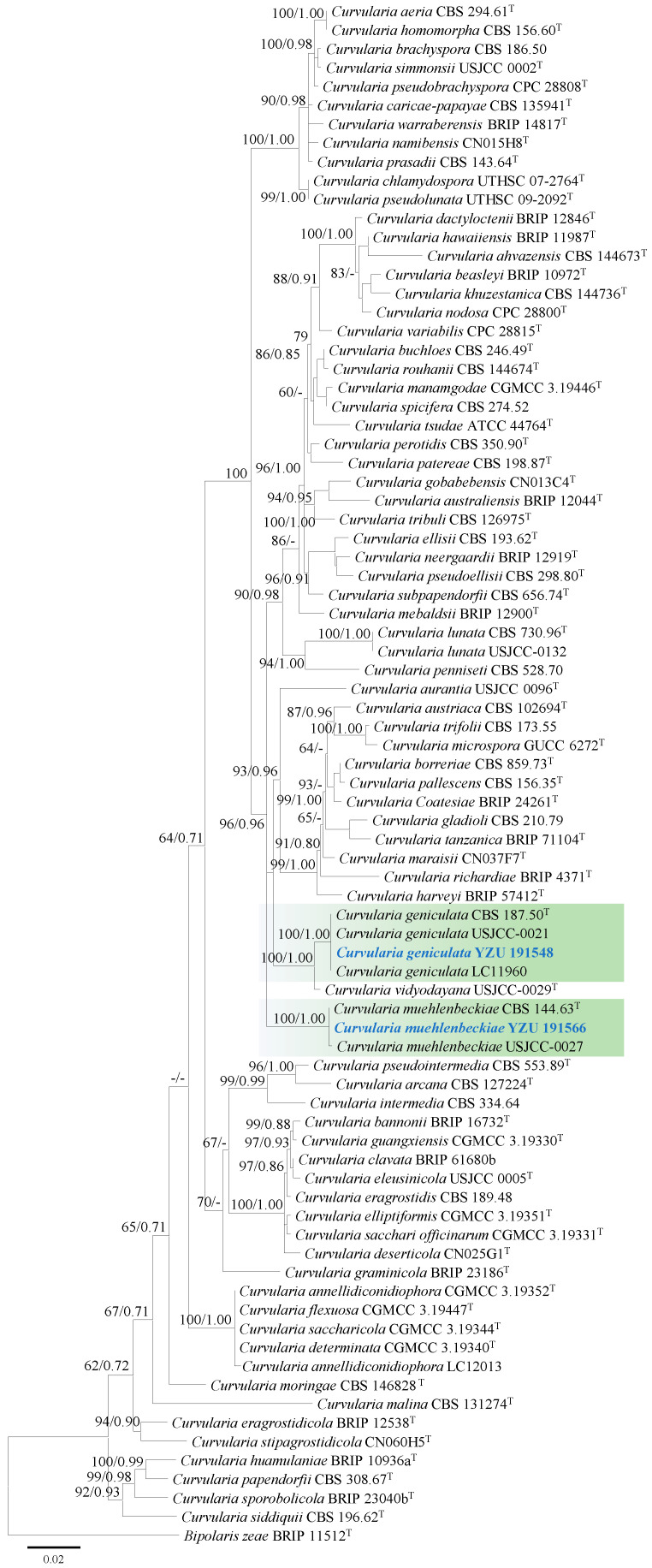
Maximum likelihood phylogenetic tree of *Curvularia* spp. inferred from the combined datasets of the ITS and *GAPDH* gene sequences. *Bipolaris zeae* (BRIP 11512) was used as the outgroup taxon. Bootstrap values and Bayesian posterior probabilities are indicated on the branches. Ex-type strains are indicated with ‘T’. Strains from this study are indicated in blue bold.

Strain YZU 191548 clustered with the ex-type strain BRIP 57412 and representative strains USJCC-0021 and LC11960 of *Curvularia geniculata*, with strong support (bootstrap value = 100%, Bayesian posterior probability = 1.00; Figure 2). *Curvularia vidyodayana* (USJCC-0029) formed a sister clade to *Curvularia geniculata*. Strain YZU 191566 grouped with the ex-type strain CBS 144.63 and representative strain USJCC-0027 of *C. muehlenbeckiae*, also supported by a bootstrap value of 100% and a Bayesian posterior probability of 1.00 (Figure 2).

Strains YZU 211044, YZU 201358, and YZU 211038 showed high similarity (99–100%) to *Fusarium* species based on BLASTn searches. Phylogenetic analysis of *Fusarium* spp. was conducted using concatenated sequences of ITS, *TUB2*, *TEF1*, and *RPB2*. A total of thirty-six strains (including the three from this study) were included in the analysis. The best-fit nucleotide substitution models for each gene were as follows: TIM2e+G4 for ITS and *RPB2*, TIM2e+I+G4 for *TEF1*, and TN+F+G4 for *TUB2*.

**Figure 3:**
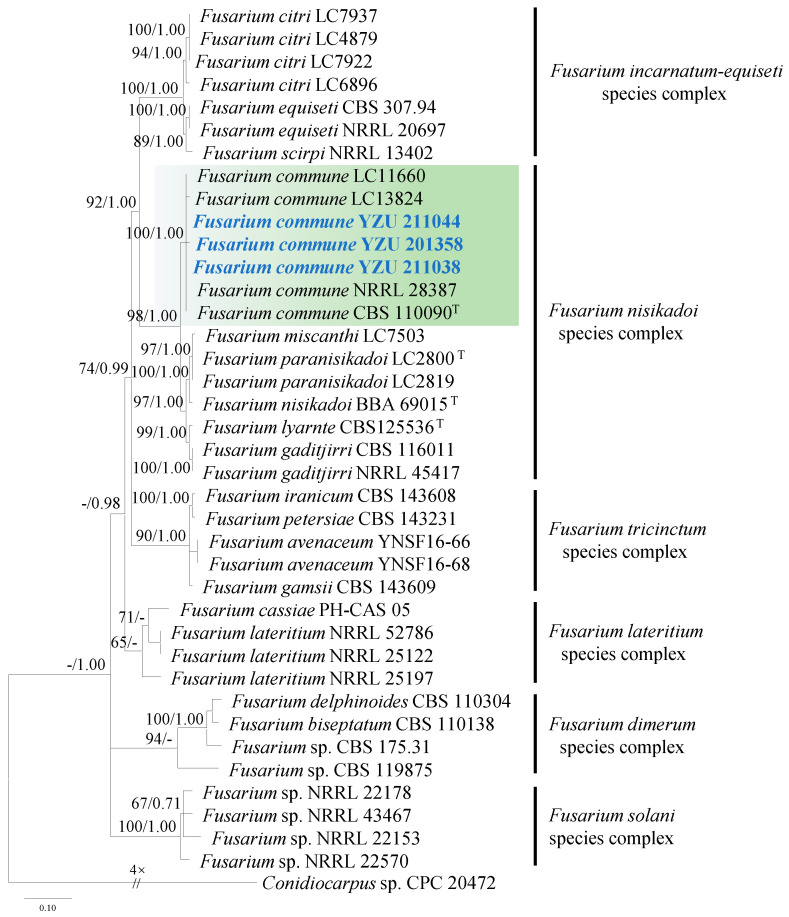
Maximum likelihood phylogenetic tree of *Fusarium* spp. inferred from the combined datasets of the ITS, *TUB2*, *TEF1*, and *RPB2* gene sequences. *Conidiocarpus* sp. (CPC 20472) was used as the outgroup taxon. Bootstrap values and Bayesian posterior probabilities are indicated on the branches. Ex-type strains are indicated with ‘T’. Strains from this study are indicated in blue bold.

The three strains (YZU 211044, YZU 201358, and YZU 211038) clustered with the ex-type strain CBS 110090 and representative strains LC11660, LC13824, and NRRL 28387 of *Fusarium commune* (*F. nisikadoi* species complex), with strong support (bootstrap value = 100%, Bayesian posterior probability = 1.00; Figure 3).

**Figure 4:**
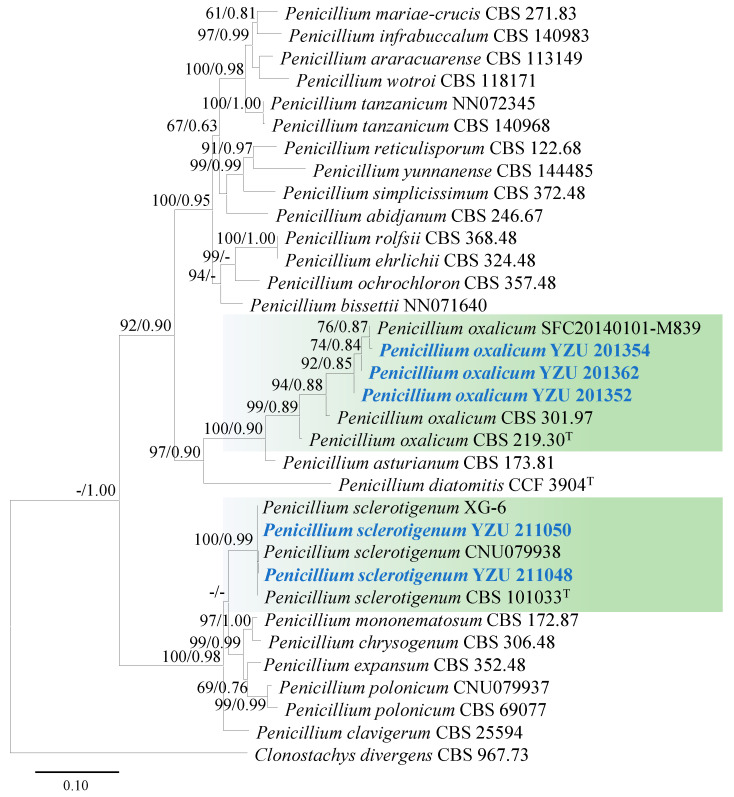
Maximum likelihood phylogenetic tree of *Penicillium* spp. inferred from the combined datasets of the ITS, *TUB2*, *CAL*, and *RPB2* gene sequences. *Clonostachys divergens* (CBS 967.73) was used as the outgroup taxon. Bootstrap values and Bayesian posterior probabilities are indicated on the branches. Ex-type strains are indicated with ‘T’. Strains from this study are indicated in blue bold.

For the identification of five strains (YZU 201352, YZU 201354, YZU 201362, YZU 211048, and YZU 211050) of *Penicillium* species, concatenated sequences of ITS, *TUB2*, *CAL*, and *RPB2* were used for phylogenetic analysis. The best-fit models for these genes were TIM2e+I+G4, K2P+G4, TNe+I+G4, and TNe+G4, respectively. A total of twenty-nine strains (including five strains from this study) were used for the phylogeny. Based on the generated phylogenetic tree, strains YZU 201354, YZU 201362, and YZU 201,352 were clustered with the ex-type strain (CBS 219.30) and representative strains (SFC20140101-M839, CBS 301.97, and CBS 219.30) of *P. oxalicum*, supported by a bootstrap value of 99% and a Bayesian posterior probability of 0.89 (Figure 4). Strains YZU 211048 and YZU 211050 were grouped together with ex-type strain (CBS 101033) and representative strains (XG-6 and CNU079938) of *P*. *sclerotigenum*, supported by a bootstrap value of 100% and a Bayesian posterior probability of 0.99 (Figure 4).

### 3.3. Taxonomy: Curvularia geniculata YZU 191548

**Figure 5:**
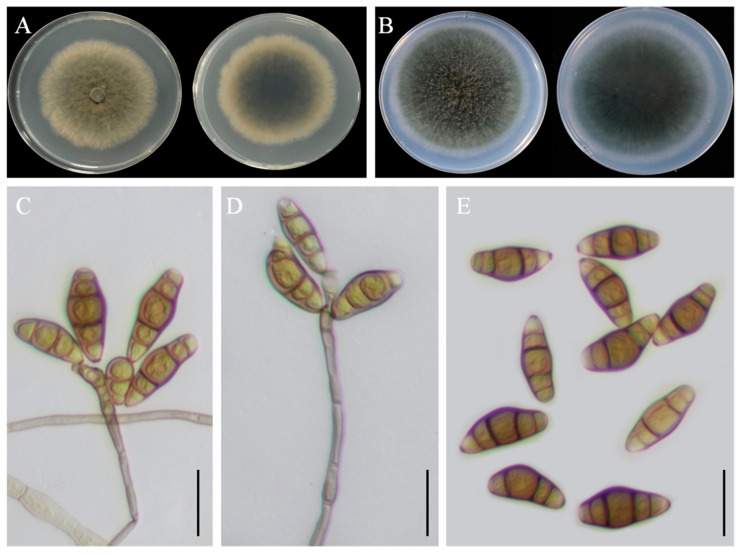
Morphological characteristics of *Curvularia geniculata* YZU 191548. (**A**) Colony on PDA for 7 days at 25 °C; (**B**) colony on PCA for 7 days at 25 °C; (**C**–**E**) conidiophores and conidia. Scale bars, (**C**–**E**) = 20 µm.

Colony morphology: On PDA, the colony reached approximately 63 mm in diameter after 7 days at 25 °C. It was olivaceous brown with an uneven margin, and the reverse side was dark in the center, surrounded by a yellowish margin (Figure 5A). On PCA, the colony reached 78–79 mm in diameter after 7 days at 25 °C, appeared greyish-green, and had a dark-brown reverse side (Figure 5B).

Asexual morphology: Hyphae were thin, smooth-walled to verruculose, septate, subhyaline, and measured 2.5–4 µm in diameter. Conidiophores were mononematous, septate, straight or flexuous, mostly geniculate, unbranched or slightly branched, pale brown to brown, and measured 20(–190) µm in length. Conidia were subcylindrical to ellipsoidal, with broad central cells, verruculose and guttulate, pale brown, and 3–4 distoseptate, measuring 15(–25) × 5(–12) µm (Figure 5C–E). Conidiogenous loci were terminal or intercalary, mono- or sympodially proliferating, pale brown, subcylindrical, and 3–6 µm in width. The sexual morph was not observed.

Note: Based on phylogenetic analysis using ITS and *GAPDH* gene sequences, strain YZU 191548 was identified as *Colletotrichum geniculata*. This study represents the first identification of this fungus in association with tuber rot of *Dioscorea esculenta*.

### 3.4. Taxonomy: Curvularia muehlenbeckiae YZU 191566

**Figure 6:**
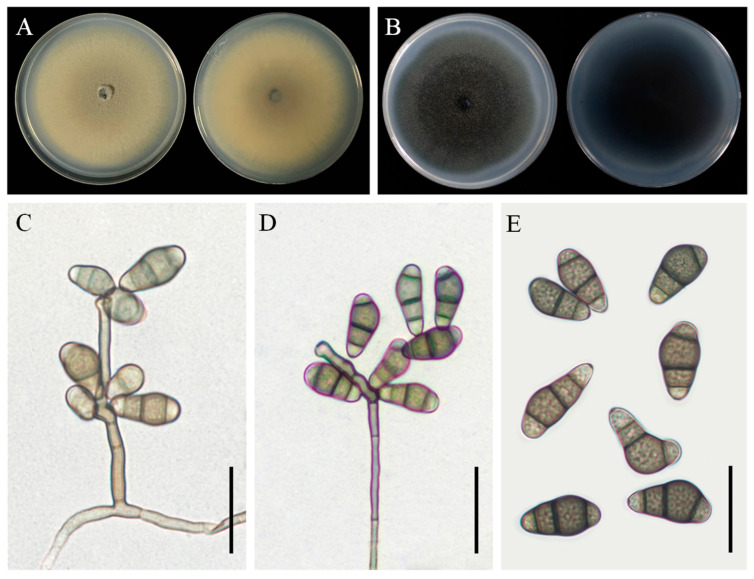
Morphological characteristics of *Curvularia muehlenbeckiae* YZU 191566. (**A**) Colony on PDA for 7 days at 25 °C; (**B**) colony on PCA for 7 days at 25 °C; (**C**–**E**) conidiophores and conidia. Scale bars, (**C**–**E**) = 20 µm.

Colony morphology: On PDA, the colony reached 85–86 mm in diameter after 7 days at 25 °C. It was olivaceous to yellowish-brown, with cottony aerial mycelium and a yellowish-brown reverse side (Figure 6A). On PCA, the colony reached 84–86 mm in diameter after 7 days at 25 °C. It was dark brown with a white margin, and the reverse side was dark (Figure 6B).

Asexual morphology: Hyphae were subhyaline to pale brown, branched, septate, and measured 1.5–5 µm in diameter. Conidiophores occurred singly or in groups, were septate, straight or flexuous, smooth-walled, mononematous, and sometimes branched, with lengths ranging up to 300 µm. Conidia were slightly verruculose, with middle cells unequally enlarged, subhyaline to pale brown, ellipsoidal to obovoid, and 3–4 distoseptate, measuring 14(–19) × 7(–9) µm (Figure 6C–E). Conidiogenous cells were smooth-walled, terminal or intercalary, sympodially proliferating, pale brown, and subcylindrical to swollen, measuring 4.5–14 µm in width. The sexual morph was not observed.

Note: Based on phylogenetic analysis using ITS and *GAPDH* gene sequences, strain YZU 191566 was identified as *Colletotrichum muehlenbeckiae*. This species was identified for the first time in association with tuber rot of *Dioscorea esculenta*.

### 3.5. Taxonomy: Fusarium commune YZU 201358

**Figure 7:**
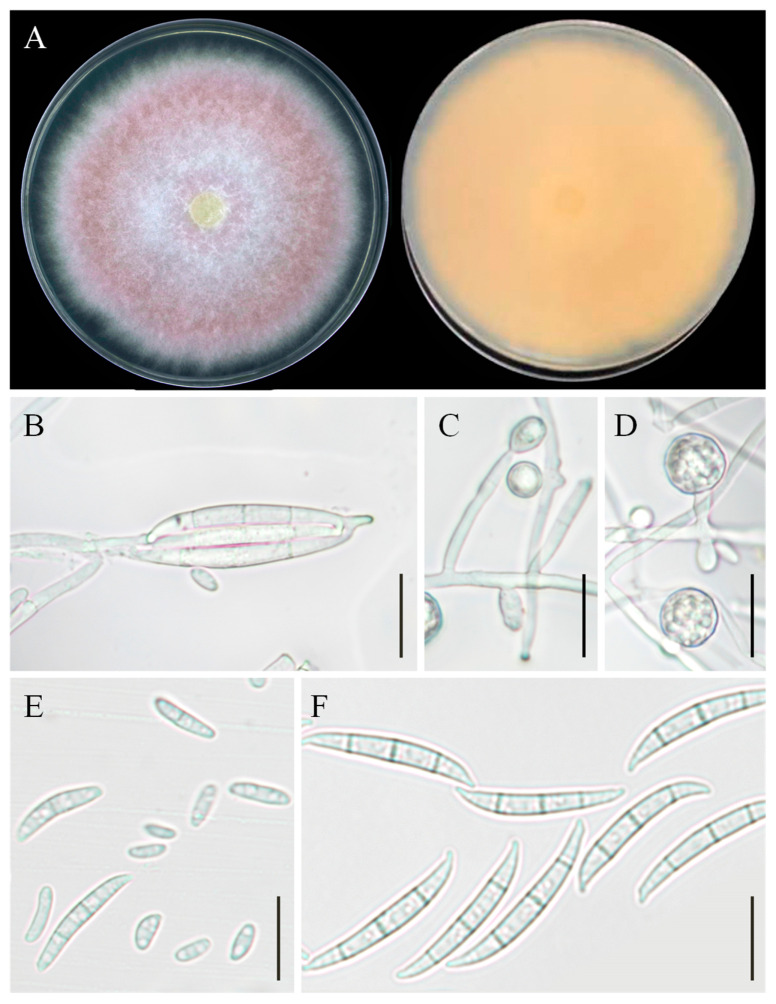
Morphological characteristics of *Fusarium commune* YZU 201358. (**A**) Colony on PDA for 7 days at 25 °C; (**B**,**F**) macroconidia; (**E**) microconidia; (**C**,**D**) chlamydospores. Scale bars, (**B**–**F**) = 30 µm.

Colony morphology: On PDA, the colony reached 88–89 mm in diameter after 7 days at 25 °C. The front side was pink with a white margin and center, and the reverse side was yellow (Figure 7A).

Asexual morphology: Macroconidia were abundant, elongate, and straight, measuring 3 × 35 μm, with 3–4 septa. The apical cells were tapered and curved, and the basal cell was developed, foot-shaped, 8–13 μm long, elongated, and barely to distinctly notched, measuring 9–14 μm in length (Figure 7B,F). Microconidia were fusiform to obovoid, measuring 5–16 × 2.5 μm, with 0–1 septa (Figure 7E). Chlamydospores were globose or subglobose (Figure 7C,D). Conidiogenous cells were monophialidic or polyphialidic, formed from aerial mycelium, measuring 6–30 × 3–3.5 μm in size.

Note: Strain YZU 201358 was identified as *Fusarium commune* based on phylogenetic analysis using concatenated sequences of the ITS, *TUB2*, *TEF1*, and *RPB2* regions. This is the first report of *F. commune* being associated with tuber rot of *Dioscorea esculenta*.

### 3.6. Taxonomy: Penicillium sclerotigenum YZU 211050

**Figure 8:**
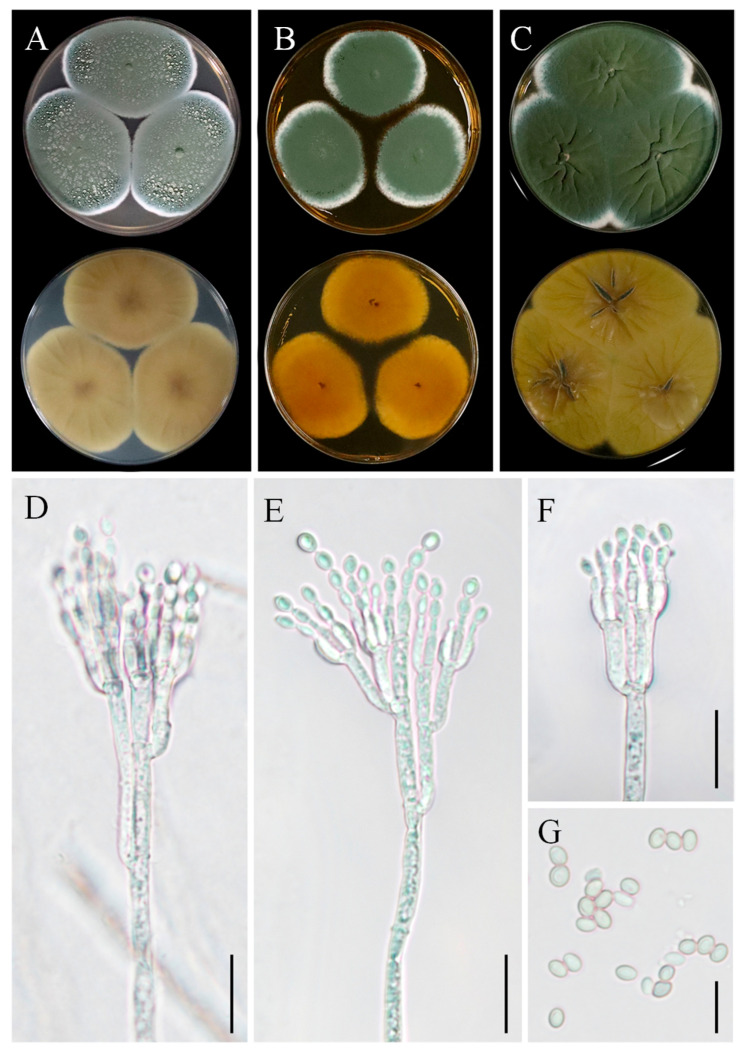
Morphological characteristics of *Penicillium sclerotigenum* YZU 211050. (**A**) Colony on CYA; (**B**) colony on MEA; (**C**) colony on YES; (**D**–**F**) conidiophores and conidia; (**G**) conidia; scale bars, (**D**–**F**) = 15 µm, (**G**) = 10 µm.

Colony morphology: On CYA, colonies were velvety, with an aniline yellow reverse side, displaying a darker yellow center (Figure 8A). On MEA, colonies were floccose, devoid of exudates, with moderate sporulation. The reverse side was reddish-yellow to orange (Figure 8B). On YES, colonies were floccose, with moderate to abundant sporulation, dull green in color, and the reverse side was light yellow with a dark-yellow center (Figure 8C).

Asexual morphology: Conidiophores were borne from surface hyphae. On MEA, stipes were mostly straight, measuring 200–600 µm in length, smooth-walled, rough-walled, biverticillate, and quadriverticillate. Metulae were cylindrical, measuring 15–25 µm in length. Phialides were smooth-walled, measuring 8–12 µm in length. Conidia were ellipsoidal, measuring 4–5 µm × 2.5–3.5 µm (Figure 8D–G).

Note: Morphologically, this fungus was similar with the previous description of *P*. *sclerotigenum* [39]. Phylogenetically, strain YZU 21050 clustered with reference strains of *P*. *sclerotigenum*. This is the first report of this fungus being associated with tuber rot of *D*. *esculenta*.

### 3.7. Taxonomy: Penicillium oxalicum YZU 201352

**Figure 9:**
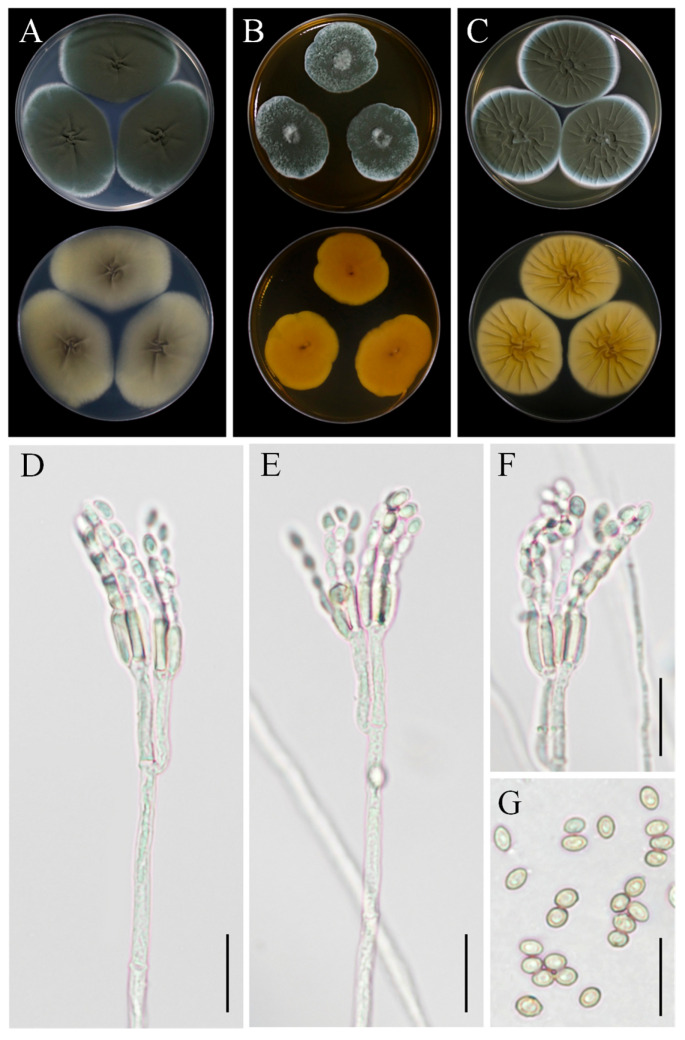
Morphological characteristics of *Penicillium oxalicum* YZU 201352. (**A**) Colony on CYA; (**B**) colony on MEA; (**C**) colony on YES; (**D**–**F**) conidiophores and conidia; (**G**) conidia; scale bars, (**D**–**F**) = 30 µm, (**G**) = 15 µm.

Colony morphology: On CYA, colonies were light yellow to dark yellow on the reverse side, with a dark-brown center and a whitish margin. The colonies were velutinous to floccose, with abundant sporulation (Figure 9A). On MEA, the colony margin was velutinous to lanose (Figure 9B). On YES, colonies were sulcate, whitish to light green, with the reverse side yellow to light greenish-yellow (Figure 9C).

Asexual morphology: Conidiophores were terminal, branched, typically biverticillate with 2–3 adpressed metulae or monoveticillate. Stipes were smooth, measuring mostly 75–400(–550) × 2.5–3.0 μm. Metulae were 11–25 × 2.5–4.5 μm. Phialides were cylindrical with a distinct collula, 2–6 per metula, measuring 8.5–15(–19) × 2.0–4.0 μm. Conidia were finely roughened, ellipsoidal, measuring 4–5.5 × 2.7–3.5 μm (Figure 9D–G). The sexual state was not known.

Note: Based on phylogenetic analysis using four markers (ITS, *TUB2*, *CAL*, and *RPB2*), strain YZU 201352 clustered closely with strains of *P. oxalicum* (XG6, CNU079938, and CBS 101033), with high statistical support. This fungus was identified here for the first time in association with tuber rot of *Dioscorea esculenta*.

### 3.8. Pathogenicity

**Figure 10:**
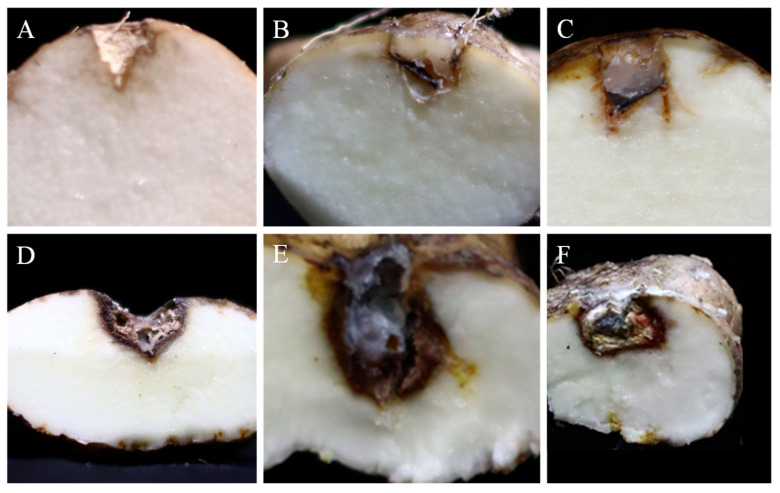
Pathogenicity tests of fungal strains from this study on Foshou yam (*Dioscorea esculenta*) tubers. (**A**) Control; (**B**) *Curvularia geniculata* YZU 191548; (**C**) *Curvularia muehlenbeckiae* YZU 191566; (**D**) *Fusarium commune* YZU 201358; (**E**) *Penicillium oxalicum* YZU 201352; (**F**) *Penicillium sclerotigenum* YZU 211050.

The pathogenicity of the five fungal species was tested on healthy Foshou yam tubers using the following strains: YZU 191548 (*C. geniculata*), YZU 191566 (*C. muehlenbeckiae*), YZU 201358 (*Fusarium commune*), YZU 201352 (*P. oxalicum*), and YZU 211050 (*P. sclerotigenum*). Lesion diameters were measured 2 weeks post-inoculation. All fungal strains induced rot in yam tubers, while those in the control group remained healthy (Figure 10). Of all the tested strains, strain YZU 201352 (*P. oxalicum*) showed significantly higher pathogenicity than the others, with mean lesion diameters reaching 22.17 mm (Table 1). No significant difference was observed among the other four strains (Table 1). The mean lesion diameters caused by these four strains were similar: 10.65 mm for YZU 191548 (*C. geniculata*), 13.31 mm for YZU 191566 (*C. muehlenbeckiae*), 13.01 mm for YZU 201358 (*Fusarium commune*), and 11.50 mm for YZU 211050 (*P. sclerotigenum*). Each fungal pathogen was successfully reisolated from the inoculated tubers and reidentified by morphological and molecular methods, which confirms Koch’s postulates.

## 4. Discussion

Foshou yam (*D. esculenta*) is an agricultural product with geographical indication in Wuxue, China. In this study, tuber rot of Foshou yam was observed in Wuxue city from 2019 to 2021. Accurate identification of the pathogen is important for disease control. This study combined morphological analysis and multi-locus phylogeny to identify the fungal pathogens causing tuber rot in *D. esculenta* to the species level. The fungal species were identified as *Curvularia geniculata*, *C. muehlenbeckiae*, *F. commune*, *P. oxalicum*, and *P. sclerotigenum* based on combined analyses of morphology and multi-locus phylogeny. In addition, pathogenicity tests revealed differential pathogenicity among these species, with *P. oxalicum* showing the highest aggressiveness. To our knowledge, this is the first study to systematically characterize fungal pathogens involved in tuber rot in Foshou yam (*D. esculenta*).

In China, *Neoscytalidium dimidiatum* has recently been reported to cause dieback in *D*. *esculenta* [40]. In our study, three different genera (*Curvularia*, *Fusarium*, and *Penicillium*) were involved in the tuber rot of *D. esculenta*. The genus *Curvularia* contains various species, including saprophytes, endophytes, and pathogens. In this study, two species in this genus, *C. geniculata* and *C. muehlenbeckiae*, were isolated from yam tubers as pathogens. *Curvularia geniculata* has been reported on many different hosts, primarily plants in the Poaceae family. *Curvularia muehlenbeckiae* is a species first isolated from *Muehlenbeckia* sp. [41]. Later, this fungus was found on different hosts, including *Cunninghamia lanceolata*, *Oryza* sp., *Saccharum officinarum*, *Sorghum* spp., and *Zizania latifolia* [41,42,43,44,45,46]. According to the Fungus-Host database (https://fungi.ars.usda.gov/), *C. eragrostidis* has been found on *Dioscorea* spp. in Brazil. The present study expands the host range of *C. geniculata* and *C. muehlenbeckiae*. In addition, these two species showed similar aggressiveness toward their host, Foshou yam. *Fusarium commune* is one of the causal agents of fusarium wilt. This species can cause root rot disease in some plants, such as tomato, soybean, sugarcane, and horseradish [47,48,49,50]. In China, *F. commune* has frequently been found as the causal agent of fusarium wilt in *Eleocharis dulcis* [51]. This species has not previously been reported from *Dioscorea* spp. Our study revealed that *F. commune* is associated with tuber rot in Foshou yam (*D. esculenta*) in China. In this study, *Penicillium oxalicum* and *P. sclerotigenum* were also identified as pathogens in *D. esculenta*, with *P. oxalicum* showing stronger pathogenicity than *P. sclerotigenum*. According to the Fungus-Host database (https://fungi.ars.usda.gov/), these two species have been previously reported on *Dioscorea* sp. and *D. batatas*, respectively. Additionally, it has been reported that *P. polonicum* and *P. sclerotigenum* are associated with blue mold of yam (*D. batatas*) in Korea [39].

In conclusion, this study isolated and characterized five fungal species associated with tuber rot of *D. esculenta* in China. Their roles as causal pathogens of the disease were confirmed by pathogenicity tests. Since all five fungal species have not been previously reported in *D. esculenta*, increased attention and control efforts are needed to manage these new tuber rot pathogens in the future. Moreover, as plant diseases are dynamic and complex processes, further investigation is needed into additional factors contributing to infections in Foshou yam tubers in the field (e.g., soil conditions and plant–microbe interactions), as well as potential unknown pathogens.

## Figures and Tables

**Table 1:** Lesion diameters on Foshou yam (*Dioscorea esculenta*) tubers caused by different fungal species.

Fungal Strains	Measurement of Lesion (mm) *
*Curvularia geniculata* YZU 191548	10.65 ± 0.69 b
*Curvularia muehlenbeckiae* YZU 191566	13.31 ± 0.41 b
*Fusarium commune* YZU 201358	13.01 ± 0.36 b
*Penicillium oxalicum* YZU 201352	22.17 ± 2.01 a
*Penicillium sclerotigenum* YZU 211050	11.50 ± 0.68 b

* Data are presented as mean ± standard error (SE). Different lowercase letters indicate significant differences (*p* < 0.05).

## Data Availability

The nucleotide sequences generated in this study were deposited in GenBank database.

## Supplementary Materials

The following supporting information can be downloaded at https://www.mdpi.com/article/10.3390/jof11050380/s1, Table S1: GenBank accession numbers of fungal species used for phylogenetic analyses.
